# Real-world study of phakic refractive lens for correction of high myopia

**DOI:** 10.1186/s40662-024-00423-z

**Published:** 2025-02-02

**Authors:** An-Peng Pan, Xu Shao, Yi-Ke Li, Zi-Yue Li, Qiong Yan, Wei-Yang Sun, A-Yong Yu

**Affiliations:** 1 National Clinical Research Center for Ocular Diseases, Eye Hospital, Wenzhou Medical University, 270 Xueyuan West Road, Wenzhou, 325027 Zhejiang Province China; 2 Hangzhou Xihu Zhijiang Eye Hospital, 366 Xiangshan, Hangzhou, 310008 China

**Keywords:** Phakic refractive lens, High myopia, Real-world study, Ophthalmic viscosurgical device-free

## Abstract

**Background:**

To assess the safety and efficacy of phakic refractive lens (PRL) implantation for correcting high myopia, as well as an ophthalmic viscosurgical device-free (OVD-free) method for PRL implantation.

**Methods:**

In this real-world prospective study, consecutive patients implanted with PRL in one or both eyes were enrolled. Based on the surgical techniques used, the eyes were divided into the OVD-free method group and the conventional method group. The patients were examined 2 h after surgery and were scheduled for follow-up at 1 day, 1 week, 1 month, 3 months, 6 months, and 12 months. The corrected distance visual acuity (CDVA), uncorrected distance visual acuity (UDVA), the manifest refraction spherical equivalent (MRSE), intraocular pressure (IOP) and lens vault were assessed postoperatively. Corneal endothelial cell density (ECD) was measured at the 3- and 12-month postoperative visits.

**Results:**

Fifty-seven consecutive patients (108 eyes) were enrolled. At the 3-month postoperative visit, both mean UDVA and CDVA were significantly improved after PRL implantation (0.19 ± 0.21 and 0.01 ± 0.14 logMAR) with efficacy index and safety index of 0.92 and 1.30, respectively. None of the eyes had any loss of CDVA. The percentage of eyes within ± 0.50 D and ± 1.00 D of target refraction was 58% and 83%, respectively. Mean MRSE changed from − 14.49 ± 4.22 D, preoperatively, to − 1.22 ± 1.26 D at 1 day (*P* < 0.001) and remained stable thereafter. Mean endothelial cell loss was 11.3%, 9.6%, respectively, at 3 and 12 months, with no significant difference between the two follow-ups (*P* = 0.395). Fifty-nine eyes received the OVD-free method, and 49 eyes received the conventional method. The OVD-free method demonstrated a significant reduction in the incidence of early acute IOP elevations (28.8% vs. 53.1%, *P* = 0.022) compared to the conventional method. The difference of initial endothelial cell loss (9.4 ± 14.2% vs. 13.6 ± 14.6%) between the two groups trended toward significance (*P* = 0.056). In both groups, no other major complications were observed up to 12-month follow-up.

**Conclusions:**

PRL implantation was a safe, efficient, predictable and stable method for correcting high myopia. The potential of lower incidence of early acute IOP elevations makes the OVD-free method a promising alternative to the conventional method.

*Trail registration*: Chinese Clinical Trial Registry, ChiCTR2100043600. Registered on 23 February 2021, https://www.chictr.org.cn/showproj.html?proj=122229

## Background

Over the past decade, the epidemic of myopia has been well recognized worldwide and was found to be most prevalent in East and Southeast Asia [1, 2]. As predicted by Holden et al. [3], the global prevalence of myopia will be 49.8% of the world’s population, with 9.8% being highly myopic by 2050. This “myopia boom” will be paralleled by an increasing demand for surgical correction of myopia.

Posterior chamber phakic intraocular lens (pIOL) implantation has become an efficient and safe alternative for the treatment of moderate to high myopia when corneal refractive surgery is contraindicated or less favorable [4–6]. Currently, implantable collamer lenses (ICL, STAAR Surgical, Monrovia, CA, USA) are the most popular pIOLs worldwide to correct myopia up to − 18.00 diopters (D) [6–8]. For extremely high myopia (− 18.00 D or less), the only pIOL available in China was the phakic refractive lens (PRL, Haohai Biological Technology, Shanghai, China), which has a wider range of myopia correction up to − 30.0 D. This pIOL was originally created by Fyodorov in 1987 and the lens design was refined to achieve the desired surgical results in a safe manner [9]. In China, clinical trials for the registration certificate of the current version of PRL were launched in 2006, and it received approval from the China Food and Drug Administration (CFDA, or now NMPA) for the correction of myopia in December 2009. Unlike the ICL, the PRL was designed to “float” freely within the aqueous humor without contacting the anterior surface of the crystalline lens or applying pressure on the ciliary sulcus [9, 10]. The safety and efficacy of PRL implantation for myopia correction has been reported in previous prospective, single-center, case series [11–14]. However, the majority of these studies had small sample sizes (20 eyes for Koivula et al. [12], 50 eyes for Jongsareejit [13], 53 eyes for Donoso et al. [14]). Verde et al. [11] reported favorable outcomes for a series of 90 eyes, however, only a small number of eyes with extremely high myopia were included (a mean preoperative myopia of − 11.90 ± 5.00 D). In China, the promising outcomes of PRL implantation accompanied with the growing surgical demand of myopia correction have promoted an increasing performance of PRL implantation, especially for extremely high myopia, which may be riskier due to the particularly fragile zonule [9, 15, 16]. This has raised concerns about the actual surgical outcomes and adverse events of PRL implantation in real-world practice. Meanwhile, efforts should be made to explore safer surgical methods for PRL implantation to minimize potential complications such as early acute intraocular pressure (IOP) elevation secondary to viscoelastic retention [9, 17]. The ophthalmic viscosurgical device-free (OVD-free) method of ICL implantation has been proposed previously with promising results and was considered a safe alternative to the conventional surgical method by eliminating complications associated with ophthalmic viscoelastic devices [18–21]. Meanwhile, a similar OVD-free method for the implantation of implantable phakic contact lenses (IPCL, Care Group Sight Solution, India) has also been reported, offering an additional benefit in avoiding the potential postoperative IOP spike [22]. However, the safety and efficacy of the OVD-free method for PRL implantation has not yet been evaluated.

The purpose of this study was to evaluate the safety, efficacy, and complications associated with PRL implantation for high myopia correction in a larger patient cohort. Additionally, the outcomes of the OVD-free method for PRL implantation were also investigated.

## Methods

### Patients

This real-world prospective study reviewed medical records from patients who attended a public welfare project—Hundreds of Bright Eyes—started by the Zhejiang Women and Children’s Foundation, Zhejiang Guangming Charity Foundation, and Hangzhou Aijinglun Technology Co., Ltd.

Ethics approval was obtained from the institutional review board of the Eye Hospital and School of Ophthalmology and Optometry, Wenzhou Medical University (2020-212-K-194), and the study was carried out in accordance with the tenets of the Declaration of Helsinki. Written informed consent was obtained from all participants.

This study enrolled a series of consecutive patients of the Hundreds of Bright Eyes project from February 2021 to July 2021 who fulfilled the inclusion criteria: age between 20 and 50 years; myopic patients implanted with PRL in one or both eyes. Patients were excluded if they had a history of any other ocular trauma or surgery, any other unstable ocular diseases that adversely affect vision (such as keratitis, glaucoma, uveitis, lens dislocation or subluxation, etc.)

### PRL features

**Fig. 1:**
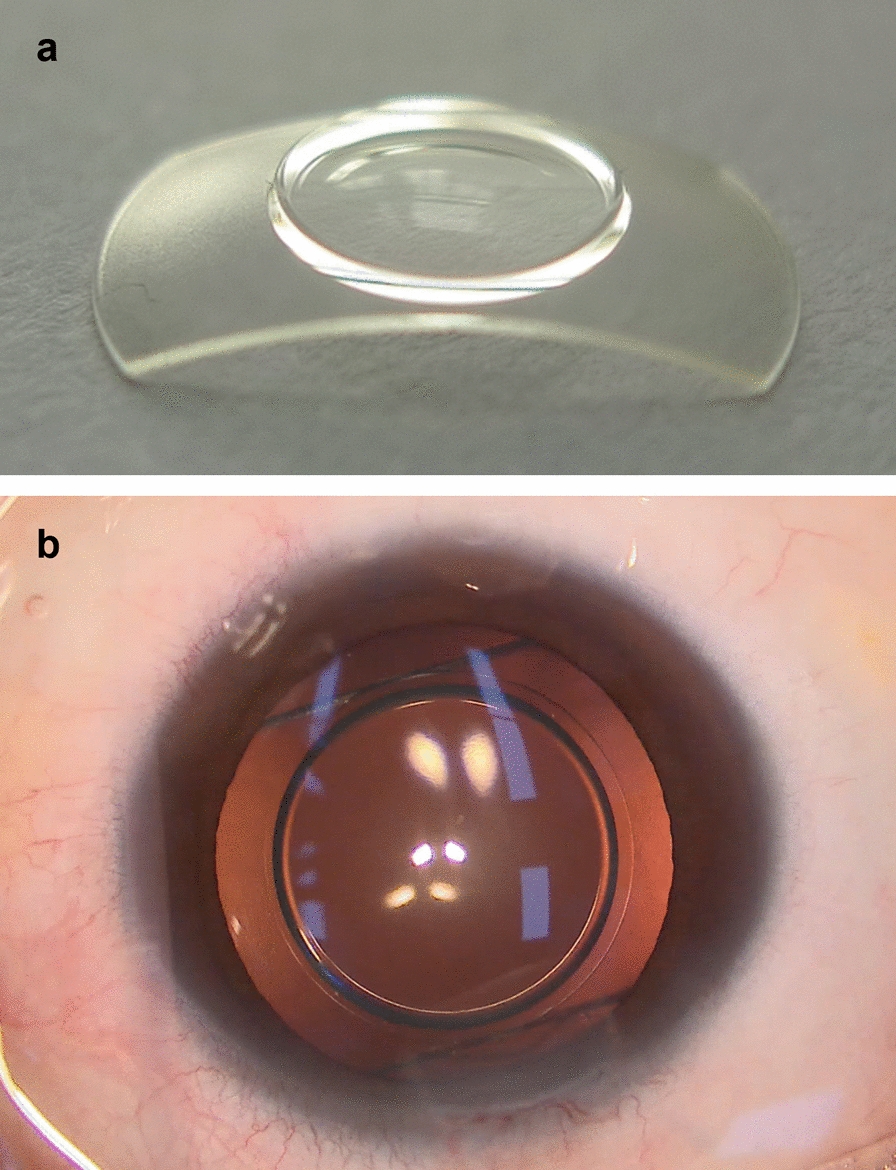
Phakic refractive lens models and intraoperative image. **a** An image of the phakic refractive lens (PRL, Haohai Biological Technology, Shanghai, China): model BK 108, optic zone 4.5–5.0 mm, width 6.0 mm, and length 10.8 mm; model BK 113, optic zone 4.5–5.0 mm, width 6.0 mm, and length 11.3 mm; **b** An intraoperative image demonstrates the successful placement of the PRL in the posterior chamber

Two models of PRL were used: BK 108 and BK 113 with a total diameter of 10.8 mm and 11.3 mm, respectively (Fig. 1a). The recommendation for size selection was as follows: model BK 108 for myopic eyes with white-to-white ≤ 11.00 mm, model BK 113 for myopic eyes with white-to-white > 11.0 mm. This lens is designed to float freely in the aqueous humor and is intended for implantation in the posterior chamber (Fig. 1b). Lens power calculation was performed using a nomogram provided by the manufacturer, which was based on the preoperative manifest refraction spherical equivalent (MRSE), the average keratometry (K), and anterior chamber depth (ACD), to achieve the target postoperative refraction.

### Preoperative data collection

The preoperative corrected distance visual acuity (CDVA), uncorrected distance visual acuity (UDVA), MRSE, corneal endothelial cell density (ECD) and IOP were recorded for all patients. The ocular biometric parameters were collected: axial length (AL), K, ACD. The parameters of the PRL (power and size) were also collected.

All patients had a complete preoperative ophthalmic examination including slit-lamp microscopy for both anterior segment assessment and dilated fundus examination. The optical coherence tomography was acquired and assessed to rule out possible unstable macular disease. The preoperative ultrasound biomicroscopy reports were reviewed to document the presence of ciliary body cysts. Neodymium:YAG laser peripheral iridotomy was performed preoperatively to create two iridotomies spaced 90° apart.

### Surgical technique

**Fig. 2:**
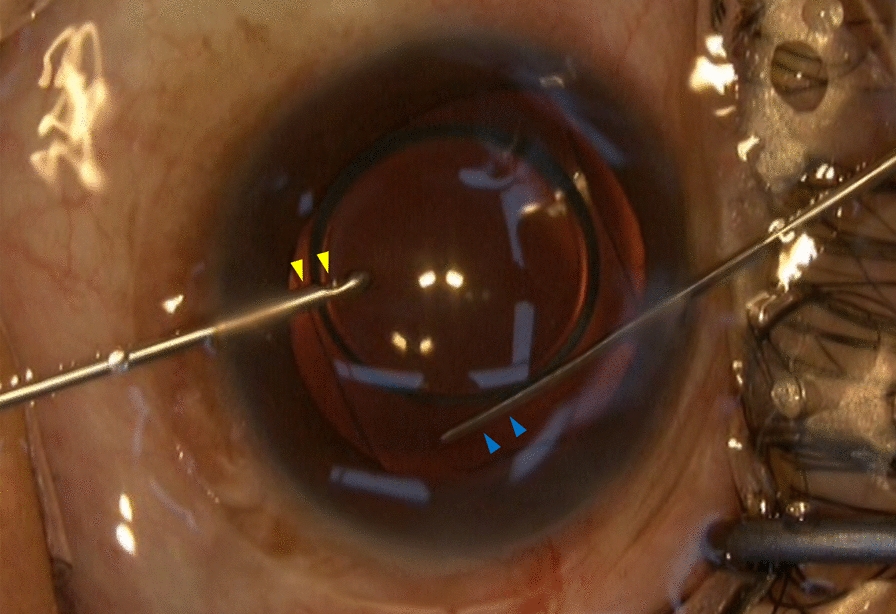
An intraoperative image of ophthalmic viscosurgical device-free method of phakic refractive lens implantation. The yellow triangles indicate the patent irrigator, the blue triangles indicate the patent manipulator, and the anterior chamber was well maintained throughout the surgical procedure

All surgeries were performed by experienced surgeons under pupil dilation with 0.5% tropicamide and 0.5% phenylephrine hydrochloride eye drops (Sinqi, Shenyang, China). The PRL was loaded into a cartridge filled with an OVD (1.7% sodium hyaluronate, Bausch & Lomb, Zhengda Freedom Group, Shandong, China), then implanted using one of the two following methods, and the eyes were divided into two groups based on the method used: (1) the conventional method, a 3.0-mm temporal or superior clear corneal main incision and one side-port (1.0 mm in size) were created. The anterior chamber was filled with an OVD and the PRL was inserted through the main incision, a lens manipulator was used to place all footplates beneath the iris. Then, the remaining OVD was manually irrigated out of the anterior chamber with balanced salt solution; (2) the OVD-free method, a 3.0-mm temporal clear corneal main incision was created, a 0.5-mm side-port was used to fill and maintain the anterior chamber by a patent irrigator (Fig. 2, yellow triangles) with balanced salt solution, a 0.3-mm side-port was used for placing footplates into the posterior chamber by a patent manipulator (Fig. 2, blue triangles). The anterior chamber was expected to be well maintained throughout the surgical procedure with the continuous infusion of balanced salt solution, and there was no need to irrigate the anterior chamber at the end of the surgery as only a small amount of OVD was used to fill the cartridge for PRL loading. Other details for the OVD-free method have been described previously [18]. Intraoperative complications were reviewed for all surgeries.

Postoperatively, 0.3% tobramycin and 0.1% dexamethasone eye drops (Alcon, USA) were administered topically 4 times daily for the first week and then 0.1% fluorometholone (Santen Pharmaceutical Co. Ltd., Japan) was employed 4 times daily for the second week, 3 times daily for the third week and twice daily for the fourth week, 0.1% pranoprofen (Senju Pharmaceutical Co., Ltd., Japan) were administered topically 4 times daily for 2 weeks, 0.1% sodium hyaluronate eye drops (URSAPHARM Arzneimittel GmbH, Germany) were administered topically 4 times daily for 4 weeks.

### Postoperative follow-up

Patients were examined 2 h after surgery and were scheduled for follow-up at 1 day, 1 week, 1 month, 3 months, 6 months, and 12 months. The slit-lamp evaluation (to assess corneal edema, PRL position, pupillary block, cataract formation, and any other abnormality), visual acuity (corrected and uncorrected), IOP (measured by Non-contact tonometer) and lens vault (the distance between the anterior lens capsule and posterior surface of PRL measured by anterior segment optical coherence tomography) were assessed at each visit; the ECD was measured at 3-month and 12-month postoperative visits.

### Statistical analysis

Statistical analyses were performed using SPSS (version 25.0, IBM SPSS Statistics for Windows, Armonk, NY). The Shapiro–Wilk test was used for testing normality. All continuous variables are expressed as the mean ± standard deviation, and categorical variables are presented as percentages. Independent samples *t*-test or the Mann–Whitney U test was used for comparisons between two groups. A linear mixed model was utilized to compare the temporal variations in parameters such as IOP, MRSE, ECD, and lens vault. The LSD-t test was then employed for further pairwise analyses and intergroup comparisons. The Chi-squared test was used to compare proportions between the two groups. A *P* value of less than 0.05 indicates statistical significance.

## Results

One hundred and eight eyes of 57 consecutive patients (52 females) were included in the study. The mean age was 33.4 ± 7.6 years (range: 20 to 50 years). The mean AL was 28.68 ± 2.06 mm (range: 24.61–35.17 mm) and mean ACD was 3.09 ± 0.31 mm (range: 2.42–3.80 mm). The mean MRSE was − 14.49 ± 4.22 D (range: − 9.00 to − 27.38 D) and mean power of the PRL was − 14.14 ± 3.57 D (range: − 10.00 to − 23.75 D). Seventy-four eyes (68.5%) had a MRSE less than or equal to − 12.00 D (extremely high myopia). Seventy-two eyes (66.7%) were implanted with model BK 113, and 36 eyes (33.33%) were implanted with model BK 108. The number of eyes assessed at each follow-up were: 95 eyes (88.0%) at 2 h, 105 eyes (97.2%) at 1 day, 106 eyes (98.1%) at 1 week, 87 eyes (80.6%) at 1 month, 87 eyes (80.6%) at 3 months, 77 eyes (71.3%) at 6 months, 73 eyes (67.6%) at 12 months.

Initially, 62 eyes received the OVD-free method, however, three of them were converted to the conventional method intraoperatively due to PRL break or PRL flip. Therefore, for the postoperative comparison, 59 (54.6%) eyes were divided into the OVD-free method group, and 49 eyes (45.4%) were divided into the conventional method group. The preoperative demographic data of the two groups are summarized in Table 1. There was no significant difference between the groups with respect to age, MRSE, UDVA, CDVA, AL, IOP and PRL power. However, ACD and ECD were significantly higher in the OVD-free vs. the conventional method group (*P* = 0.028, *P* = 0.007, respectively).

**Table 1:** Preoperative demographic data for the study population

Characteristics	OVD-free method group	Conventional method group	*P*
Number of eyes	59	49	–
Age (years), mean ± SD	33.49 ± 8.00	33.76 ± 7.20	0.956^a^
Preoperative characteristics, mean ± SD			
MRSE (D)	− 14.39 ± 3.61	− 14.61 ± 4.89	0.641^a^
UDVA (logMAR)	1.36 ± 0.37	1.33 ± 0.34	0.588^a^
CDVA (logMAR)	0.14 ± 0.19	0.16 ± 0.23	0.969^a^
AL (mm)	28.54 ± 1.51	28.86 ± 2.59	0.856^a^
ACD (mm)	3.15 ± 0.30	3.02 ± 0.31	0.028^c^
ECD (cells/mm^2^)	2825.9 ± 370.1	2645.8 ± 200.5	0.007^b^
IOP (mmHg)	14.6 ± 2.0	16.1 ± 2.4	0.064^b^
PRL power (D)	− 14.06 ± 3.32	− 14.24 ± 3.88	0.797^a^

*OVD-free* = ophthalmic viscosurgical device-free; *MRSE* = manifest refraction spherical equivalent; *UDVA* = uncorrected distance visual acuity; *CDVA* = corrected distance visual acuity; *AL* = axial length; *ACD* = anterior chamber depth; *ECD* = endothelial cell density; *IOP* = intraocular pressure; *PRL* = phakic refractive lens; *D* = diopter

^a^Mann-Whitney U test

^b^Linear mixed model

^c^Independent samples *t*-test

### Visual and refractive outcomes

The visual and refractive outcomes in 108 eyes implanted with PRL are listed in Table 2. There was a significant improvement in mean UDVA after PRL implantation (*P* < 0.001), and the UDVA stabilized at 1 week with no significant changes during the subsequent follow-up (1 week vs. 1 month to 12 months, all *P*s > 0.05). The corresponding efficacy indices (mean postoperative UDVA/mean preoperative CDVA) were 0.72, 0.84, 0.91, 0.92, 0.92 and 0.94, at 1 day, 1 week, 1 month, 3 months, 6 months and 12 months postoperatively, respectively.

**Table 2:** Visual and refractive outcomes in 108 eyes that underwent phakic refractive lens implantation for myopia correction

Parameter (mean ± SD)	Pre-op	1 day	1 week	1 month	3 months	6 months	12 months	*P*^#^
UDVA (logMAR)	1.35 ± 0.36 (n = 100)	0.32 ± 0.30 (n = 105)	0.22 ± 0.18 (n = 106)	0.18 ± 0.19 (n = 87)	0.19 ± 0.21 (n = 87)	0.18 ± 0.19 (n = 76)	0.18 ± 0.21 (n = 73)	< 0.001
Efficacy index	–	0.72	0.84	0.91	0.92	0.92	0.94	–
CDVA (logMAR)	0.15 ± 0.21 (n = 108)	0.13 ± 0.19 (n = 75)	0.04 ± 0.13 (n = 97)	0.02 ± 0.11 (n = 77)	0.01 ± 0.14 (n = 76)	0.03 ± 0.13 (n = 68)	0.02 ± 0.13 (n = 54)	< 0.001
Safety index	–	1.04	1.22	1.25	1.30	1.24	1.28	–
MRSE (D)	− 14.49 ± 4.22 (n = 108)	− 1.22 ± 1.26 (n = 75)	− 1.13 ± 0.97 (n = 101)	− 0.96 ± 1.08 (n = 79)	− .09 ± 0.99 (n = 72)	− 1.14 ± 0.93 (n = 69)	− 1.14 ± 0.97 (n = 54)	< 0.001

*Pre-op* = preoperative; *UDVA* = uncorrected distance visual acuity; *CDVA* = corrected distance visual acuity; *MRSE* = manifest refraction spherical equivalent; *D* = diopter

^#^ Preoperative to postoperative, linear mixed model

There was a significant improvement in mean CDVA after PRL implantation (*P* < 0.001), and the CDVA stabilized at 1 month with no significant changes afterward (1 month vs. 3 months to 12 months, all *P*s > 0.05). The corresponding safety indices (mean postoperative CDVA/mean preoperative CDVA) were 1.04, 1.22, 1.25, 1.30, 1.24 and 1.28, at the 1 day, 1 week, 1 month, 3 months, 6 months and 12 months postoperatively, respectively.

**Fig. 3:**
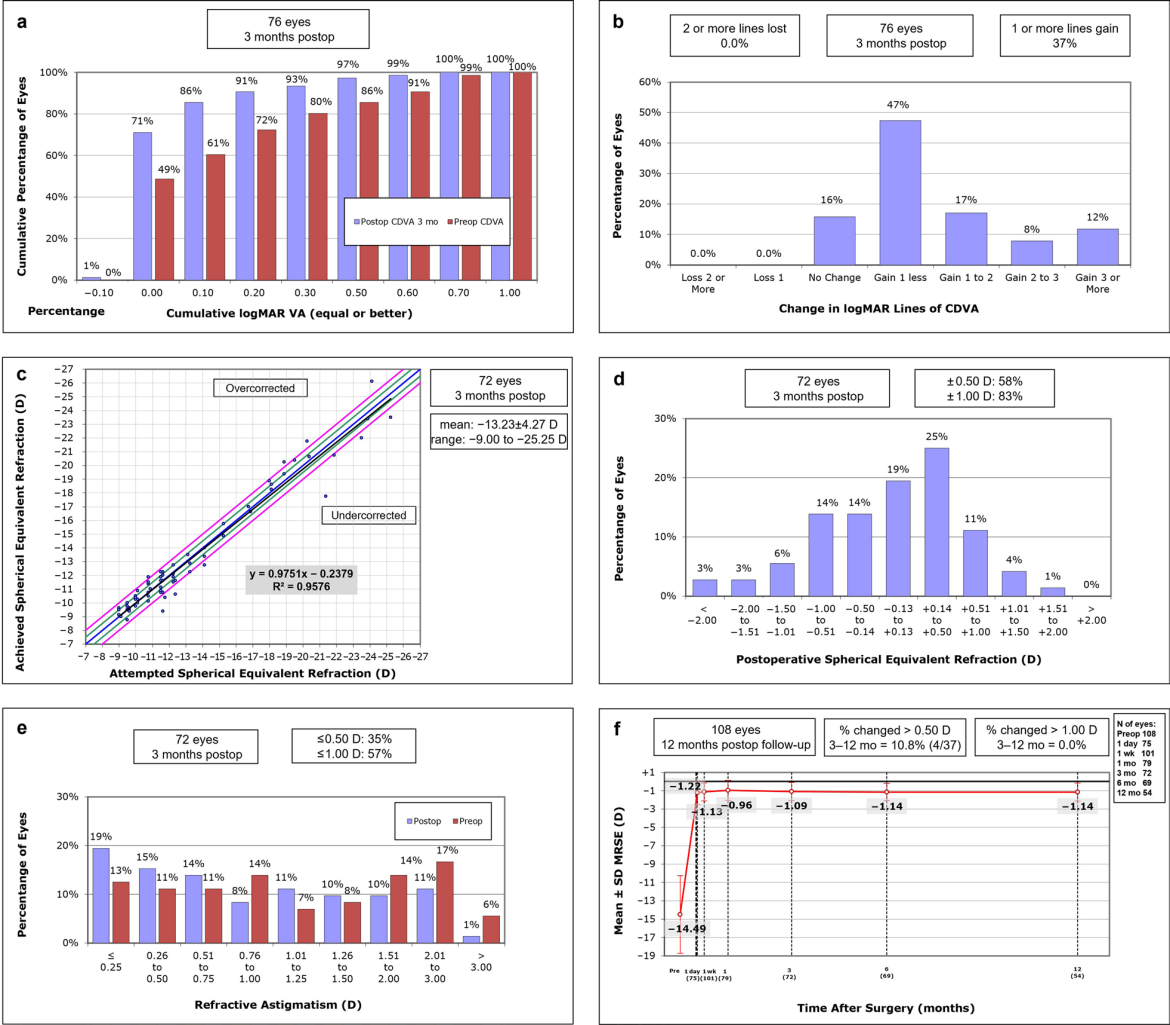
Visual and refractive outcomes after phakic refractive lens implantation. **a** Cumulative corrected distance visual acuity (CDVA) at preoperative and 3-month postoperative visits (logMAR); **b** Change in CDVA at 3-month postoperative visit compared to the preoperative visit (logMAR lines); **c** Attempted versus achieved spherical equivalent refraction, linear regression analysis and the coefficient of determination (R^2^ = 0.9899) have been calculated; **d** Postoperative spherical equivalent refractive accuracy at 3-month postoperative visit, 58% and 83% of eyes were within ± 0.50 D and ± 1.00 D of target refraction, respectively; **e** Change in refractive astigmatism between preoperative and 3-month postoperative visits; **f** The mean manifest refraction spherical equivalent (MRSE) at each visit revealed the stability of the refractive correction after phakic refractive lens implantation. The linear mixed model revealed no significant difference in the mean MRSE from 1 day to 12 months postoperatively over time (all *P* > 0.05). From 3 to 12 months postoperative visit, 10.8% (4/37) of the eyes had a change of MRSE larger than 0.5 D, but none of the eyes had a change of MRSE larger than 1.0 D. Error bars represent standard error of mean

At the 3-month postoperative visit, the mean prediction error (MPE) of refractive accuracy was − 0.09 ± 0.88D, the mean absolute error (MAE) was 0.64 D and the root mean square error (RMSE) was 0.88 D. Figure 3a compared the postoperative CDVA at the 3-month postoperative visit with preoperative visit by showing the cumulative percentage of eyes with CDVA at each logMAR line of vision in 76 eyes. At the 3-month postoperative visit, none of the eyes had any loss of CDVA, 84% of the eyes had an improvement in CDVA, and there was a gain of one line or more of CDVA in 37% of eyes (Fig. 3b). The attempted vs. achieved spherical equivalent refraction is shown in Fig. 3c, and the coefficient of determination (R^2^) was 0.9576 as calculated by linear regression analysis. Figure 3d showed the postoperative spherical equivalent refractive accuracy at the 3-month postoperative visit. The percentage of eyes within ± 0.50 D and ± 1.00 D of target refraction was 58% and 83%, respectively. The change in refractive astigmatism between the preoperative and 3-month postoperative visit is shown in Fig. 3e. As the PRL was a free-floating design, the predictable rotation of the PRL made it incapable of correcting astigmatism and therefore, as can be seen in Fig. 3e, there was no significant change in the distribution of the amount of astigmatism between the pre- and postoperative visits. Figure 3f shows statistically significant changes of the mean MRSE from − 14.49 ± 4.22 D preoperatively to − 1.22 ± 1.26 D, − 1.13 ± 0.97 D, − 0.96 ± 1.08 D, − 1.09 ± 0.99 D, − 1.14 ± 0.93 D, − 1.14 ± 0.97 D, at the 1 day, 1 week, 1 months, 3 months, 6 months and 12 months postoperatively, respectively (all *P*s < 0.001).

The stability of the refractive correction was evaluated by comparing the mean MRSE throughout the 12 months after surgery. The linear mixed model revealed no significant difference in the mean MRSE from 1 day to 12 months postoperatively over time (all *P*s > 0.05). From the 3 to 12 months postoperative visit, 10.8% (4/37) of the eyes had a change of MRSE larger than 0.5 D, and all of the changes were less than 1.0 D.

### Intraocular pressure (IOP)

The IOP increased at 2 h postoperatively to 20.0 ± 7.8 mmHg (n = 91) compared to the preoperative level (15.3 ± 2.3 mmHg, n = 108, *P* < 0.001). This elevation of IOP was maintained until 1 week after surgery (19.0 ± 4.3 mmHg, n = 106, *P* < 0.001), and thereafter, returned to preoperative level by 1 month (14.8 ± 2.6 mmHg, n = 87, *P* = 0.366). At 2 h postoperatively, 37.4% (34/91) of the eyes experienced early acute IOP elevation above 22.0 mmHg (range: 22.1–40.7 mmHg) and 30.8% (28/91) of the eyes had an increase in IOP larger than 8 mmHg (range: 8.2 to 25.1 mmHg). The percentage of eyes that had an increase in IOP larger than 8 mmHg was 10.5% (11/105) (range: 8.6 to 25.5 mmHg) at 1 day, 15.1% (16/106) (range: 8.7–20.1 mmHg) at 1 week and none at 1 month to 12 months. The clinically significant IOP elevations were closely monitored and treated with anterior chamber drainage and/or topical antiglaucoma medications as appropriate.

**Fig. 4:**
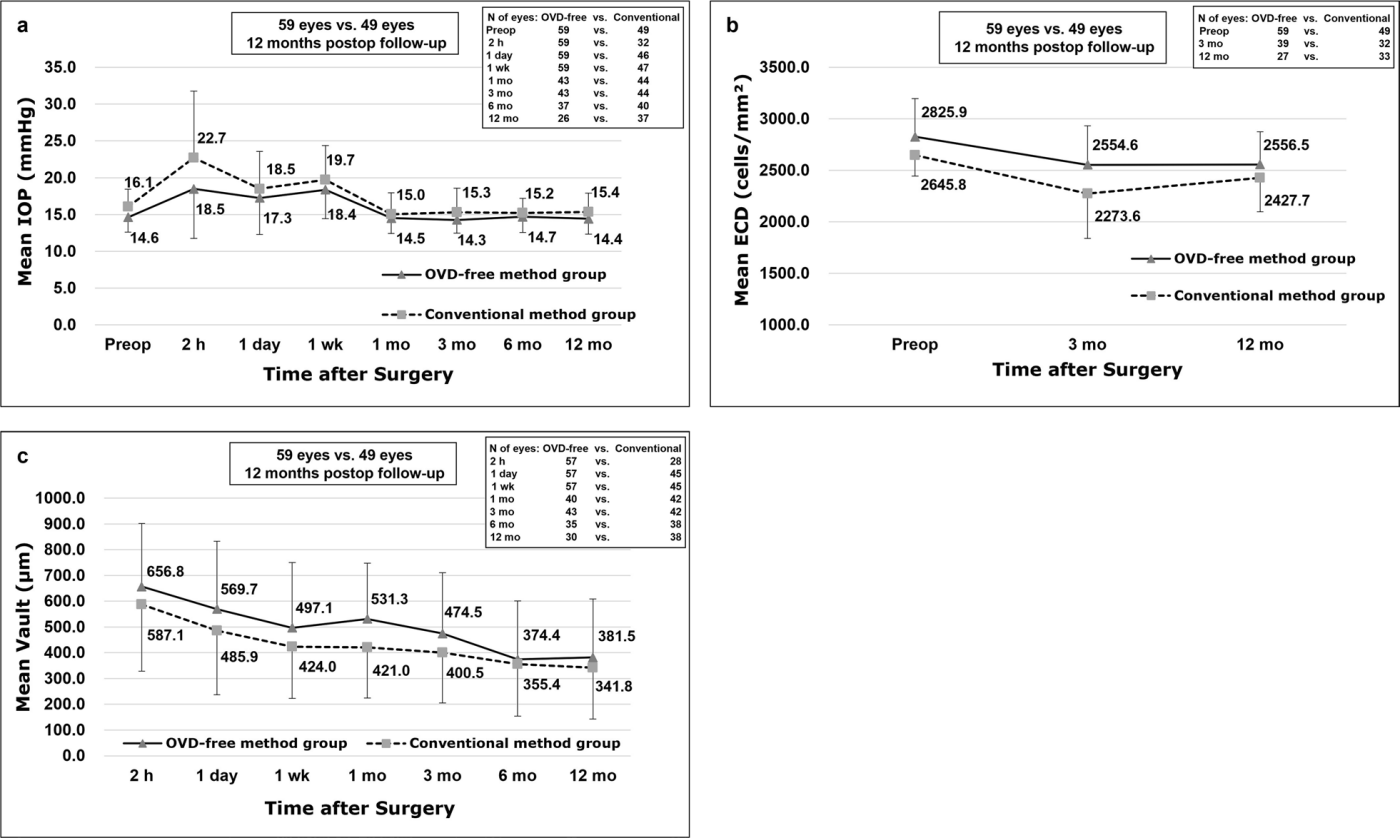
Comparison of intraocular pressure (IOP), endothelial cell density (ECD) and lens vault between two surgical methods. **a** Trends of IOP changes in the ophthalmic viscosurgical device (OVD)-free and conventional method groups; **b** Trends of mean ECD change over time after phakic refractive lens (PRL) implantation in the OVD-free and the conventional method groups; **c** Trends of lens vault changes over time after PRL implantation in the OVD-free and the conventional method groups. Error bars indicate standard error of the mean

The trend in IOP change was similar between the two groups (no interaction effect, *P* = 0.083; Fig. 4a). The differences in IOP between the two groups from 1 day to 12 months were not significant (all *P*s > 0.05). The only significant difference between the two groups was noted at 2 h postoperatively, where the IOP in the conventional method group reached 22.7 ± 9.1 mmHg, significantly higher than the OVD-free method group (18.5 ± 6.7 mmHg; *P* < 0.001). In the OVD-free method group, 28.8% (17/59) of the eyes had an IOP higher than 22.0 mmHg at 2 h postoperatively, and the incidence of acute IOP elevation was significantly higher in the conventional method group, with 53.1% (17/32) of the eyes higher than 22.0 mmHg (*P* = 0.022).

### Endothelial cell density

The ECD changed significantly from 2744.2 ± 316.9 cells/mm^2^ (n = 108) preoperatively to 2427.9 ± 425.1 cells/mm^2^(n = 71, *P* < 0.001) and 2485.7 ± 328.0 cells/mm^2^(n = 60, *P* < 0.001) at 3 months and 12 months after surgery, respectively. Measurements of ECD revealed a 11.3% (n = 71, preoperative vs. 3 months postoperative) mean endothelial cell loss at 3-month postoperative visit and 9.6% (n = 60, preoperative vs. 12 months postoperative) at 12-month postoperative visit. No statistically significant change was noted in ECD between 3 and 12 months after surgery (*P* = 0.395). At 3-month postoperative visit, 13 eyes had endothelial cell loss greater than 20%, and 30.8% (4/13) of them experienced intraoperative complications of PRL break or flip. Meanwhile, in the eight eyes known to have experienced intraoperative PRL break or flip while having ECD measurements available at 3-months postoperative visit, 50.0% (4/8) had endothelial cell loss greater than 20% (Table 3), and the incidence of notable endothelial cell loss (> 20%) was significantly higher than eyes without intraoperative complications (*P* = 0.005), which was 9.4% (9/96) (Table 4).

**Table 3:** Endothelial cell loss and visual outcomes of eyes with intraoperative complications

Eye No.	Surgical technique	Intraoperative complications	Endothelial cell loss		CDVA	
			3 months visit	12 months visit	Pre-op (logMAR)	Last visit (logMAR)
001 OS	Converted from OVD-free to Conventional	PRL break	3.9%	5.1%	0.00	− 0.08 (12 months)
004 OS	OVD-free	PRL break	27.6%	26.3%	0.22	0.15 (6 months)
008 OS	Conventional	PRL flip	NA	NA	0.00	0.00 (6 months)
009 OD	Conventional	PRL break	7.3%	17.1%	0.22	0.05 (12 months)
011 OD	Conventional	PRL break	NA	NA	0.00	0.00 (12 months)
020 OD	Converted from OVD-free to Conventional	PRL flip	48.0%	NA	0.00	− 0.08 (6 months)
023 OD	Converting from OVD-free to Conventional	PRL flip	48.0%	NA	0.15	0.05 (6 months)
026 OS	Conventional	PRL flip	NA	NA	0.70	0.30 (12 months)
028 OD	OVD-free	PRL break	NA	NA	0.30	0.05 (1 week)
042 OS	Conventional	PRL break	− 3.0%	−3.0%	0.00	− 0.08 (12 months)
049 OS	Conventional	PRL flip	42.4%	46.3%	0.10	− 0.08 (12 months)
053 OS	OVD-free	PRL break	7.6%	3.8%	0.05	0.00 (12 months)

*CDVA* = corrected distance visual acuity; *Pre-op* = preoperative; *PRL* = phakic refractive lens; *OVD-free* = ophthalmic viscosurgical device-free

**Table 4:** Notable endothelial cell loss (> 20%) of eyes without intraoperative complications

Eye No.	Surgical technique	Endothelial cell loss	
		3 months visit (%)	12 months visit (%)
004 OD	OVD-free	29.7	32.2
006 OD	OVD-free	33.3	16.8
010 OD	Conventional	47.0	21.5
021 OD	OVD-free	41.5	24.8
025 OD	Conventional	31.3	NA
025 OS	Conventional	20.9	NA
045 OD	OVD-free	52.4	NA
045 OS	OVD-free	26.8	NA
046 OD	OVD-free	21.6	NA

*OVD-free* = ophthalmic viscosurgical device-free

The trends of mean ECD changes over time after PRL implantation of the two groups are shown in Fig. 4b. At the 3 months postoperative visit, the mean endothelial cell loss was 9.4 ± 14.2% (n = 39) in the OVD-free method group and 13.6 ± 14.6% (n = 32) in the conventional method group; the difference between the two groups met a borderline level of significance (*P* = 0.056). The difference in endothelial cell loss, however, was not statistically significant at the 12-month postoperative visit with 10.5 ± 10.9% (n = 27) in the OVD-free group and 8.8 ± 10.3% (n = 33) in the conventional method group (*P* = 0.718).

### Postoperative vault

**Fig. 5:**
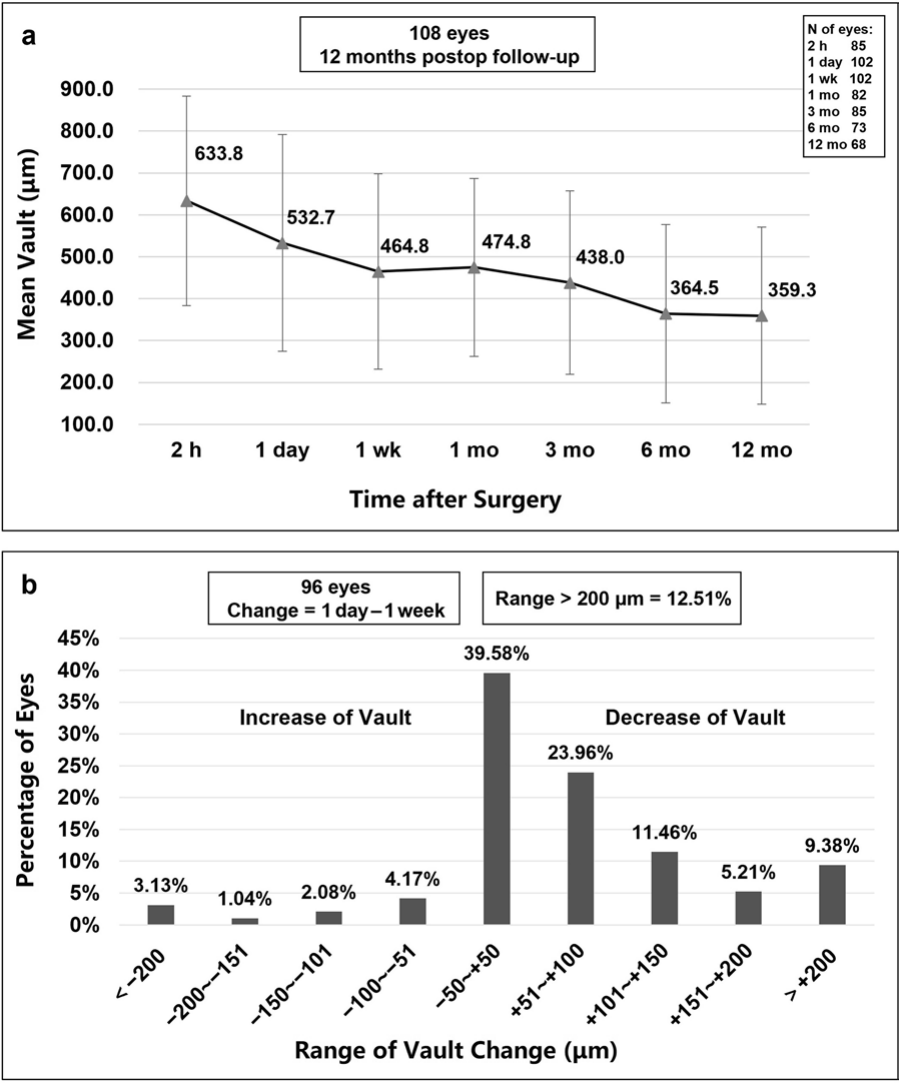
Lens vault after phakic refractive lens implantation. **a** Changes of lens vault over time. Error bars represent standard error of mean. **b** Distribution of vault change between 1 day and 1 week postoperative visits

The lens vault was defined as the distance between the posterior surface of the PRL and the anterior surface of the crystalline lens. A rapid change of the lens vault was first revealed in the short-term postoperative period (532.7 ± 258.7 μm to 464.8 ± 233.3 μm, 1 day to 1 week, *P* < 0.001), and thereafter a slow and consistent but significant decrease of the lens vault was noted (Fig. 5a). Figure 5b shows the distribution of vault change between the 1 day and 1 week postoperative visits. In the short-term postoperative period, 50.0% (48/96) of the eyes had a decrease of lens vault and 10.4% (10/96) of the eyes had an increase of lens vault, meanwhile 12.5% (12/96) of the eyes had remarkable lens vault changes (> 200 μm) from 1 day to 1 week postoperatively, either increased or decreased. The linear mixed model showed a statistically significant difference in lens vault between 2 h (633.8 ± 250.0 μm) and 1 day (*P* < 0.001), 1 day and 1 week (*P* < 0.001), 1 month (474.8 ± 212.4 μm) and 3 months (438.0 ± 219.0 μm; *P* = 0.033), 6 months (364.5 ± 212.9 μm) and 12 months (359.3 ± 211.3 μm; *P* = 0.032). No statistically significant change was noted between 1 week and 1 month (*P* = 0.506), 3 months and 6 months (*P* = 0.190).

Trends of lens vault changes over time after PRL implantation of the two groups are shown in Fig. 4c. The difference in lens vault between two groups was not statistically significant (*P* = 0.051) and no interaction effect of time × group was found between groups (*P* = 0.909). This indicated that the lens vault had the same tendency to change with time in both groups.

### Complications

Intraoperative and early postoperative complications are shown in Table 5. Complications were also compared between the two surgical methods. As shown in Table 5, the most common complication after PRL implantation was the early IOP elevations, which were lowered through appropriate management with anterior chamber drainage and/or topical antiglaucoma medications. Three eyes (4.8% of 62 eyes) were converted intraoperatively from the OVD-free method to the conventional method due to PRL break or PRL flip, and there was no statistically significant difference in the incidence of intraoperative complications such as PRL break, PRL flip, and insufficient pupil dilation between the two surgical methods (*P* = 1.000, *P* = 0.649, *P* = 1.000). Table 3 shows the visual outcomes of eyes with PRL break or PRL flip. During the early postoperative follow-up (day of surgery and 1 day postoperative visit), the incidence of postoperative complications was not significantly different between the two surgical methods.

**Table 5:** Complications in 108 eyes that underwent phakic refractive lens implantation for myopia correction

Complications	No. of eyes			*P*^#^
	OVD-free method	Conventional method	Total	
Intraoperative				
PRL break	4 (6.5% of 62)	3 (6.5% of 46)	7 (6.5% of 108)	1.000
Management (No. of eyes)	4 Using spare PRL 1 Converting from OVD-free to Conventional	3 Using spare PRL		
PRL flip	2 (3.2% of 62)	3 (6.5% of 46)	5 (4.6% of 108)	0.649
Management (No. of eyes)	2 Converting from OVD-free to Conventional and Re-implanted	3 Re-implanted		
Insufficient pupil dilation	6 (9.7% of 62)	2 (4.3% of 46)	8 (7.4% of 108)	1.000
Management (No. of eyes)	6 Intracameral epinephrine hydrochloride	2 Intracameral epinephrine hydrochloride		
Day of surgery				
Anterior chamber drainage for early IOP elevations	10 (16.9% of 59)	7 (19.4% of 36)	17 (17.9% of 95)	0.862
Topical antiglaucoma medications	0 (0% of 59)	1 (2.8% of 36)	1 (1.1% of 95)	0.426
1 day postoperative visit				
Anterior chamber drainage for IOP elevations	1 (1.7% of 59)	3 (6.5% of 46)	4 (3.8% of 105)	0.317
Topical antiglaucoma medications	17 (28.8% of 59)	9 (19.6% of 46)	26 (24.8% of 105)	0.276
1 week postoperative visit				
Topical antiglaucoma medications	21 (35.6% of 59)	10 (21.3% of 47)	31 (29.2% of 106)	0.107
1 month postoperative visit				
Topical antiglaucoma medications	0 (0% of 43)	1 (2.3% of 44)	1 (1.1% of 87)	1.000

*PRL* = phakic refractive lens; *IOP* = intraocular pressure; *OVD-free* = ophthalmic viscosurgical device-free

^#^Chi-squared test

PRL and crystalline lens contacts (lens vault less than 10 μm detected by anterior segment optical coherence tomography) were observed in two eyes of one patient (patient 55, conventional method) at 6 months postoperatively, and four eyes of two patients (patient 10 and patient 55, conventional method for both) at 12 months postoperatively, respectively. For patient 10 (age 46 years, MRSE: right eye − 22.125 D, left eye − 22.75 D, conventional method), both eyes showed a notable but stable increase in the density of crystalline lens since the 1 week postoperative visit and CDVA remained stable over the 12-month follow-up period (right eye: 0.15, 0.15, 0.15, 0.22, 0.22 logMAR, left eye: 0.22, 0.22, 0.22, 0.15, 0.15 logMAR at 1 week, 1 month, 3 months, 6 months and 12 months after surgery, respectively). Meanwhile, no decrease in CDVA and no cataract formation were observed in either eye of patient 55 (age 33 years, MRSE: right eye − 9.125 D, left eye − 9.0 D, conventional method) after detection of PRL and lens contact. At 12 months after surgery, the right eye of patient 09 (age 45 years, MRSE: right eye − 14.0 D, conventional method) and the left eye of patient 24 (age 43 years, MRSE: left eye − 17.5 D, conventional method) developed punctate lens opacities in the periphery without visual loss. Overall, no clinically significant cataract formation was found over the entire 12-month follow-up period. Mild PRL decentrations were observed in seven eyes of four patients during the 12-month follow-up period and no intervention was required. No pupillary block, retinal detachment, lens subluxation, or other major complications were observed during the 12-month follow-up period.

## Discussion

To the best of our knowledge, this study is the largest prospective series of PRL implantation to date, with a total of 108 eyes included from 57 myopic patients. The real-world clinical outcomes and complications assessment revealed that the PRL implantation was a safe, efficient, predictable and stable method for the correction of high myopia. In this study, both mean UDVA and CDVA were significantly improved, and high myopia was successfully corrected after PRL implantation. This was achieved with clinically acceptable endothelial cell loss and no major complications were observed during the 12-month of follow-up. At 3 months postoperatively, 72 eyes showed an overall satisfactory result in terms of spherical equivalent refractive accuracy with an R^2^ value of 0.9576, a MAE of 0.64 D and a RMSE of 0.88 D, but accuracy was slightly lower in higher myopic eyes (Fig. 3c). Here, 83% of eyes were within ± 1.0 D of the target refraction (MRSE: − 14.49 ± 4.22 D), a result that was comparable to those of previous studies, which ranged from 71.2% to 97.14% [11, 14, 23, 24]. However, there was a noticeable trend in the literature that the higher the degree of myopia, the lower the accuracy: 71.2% for MRSE of − 17.27 ± 4.58 D [14], 79% for MRSE of − 14.70 ± 2.65 D [23], 80% for MRSE of − 11.90 ± 5.00 D [11], 97.14% for MRSE of − 10.25 ± 3.19 D [24]. In this study, the CDVA was significantly improved after surgery with the safety indices to be 1.30, 1.24 and 1.28 at 3 months, 6 months, and 12 months postoperatively. At 3-month postoperative visit, which has the largest number of eyes available for mid-term analysis, none of the eyes had any loss of CDVA. Moreover, 84% of the eyes had an improvement in CDVA resulting from the change in the plane of correction, which may affect retinal magnification [25]. A significant number of patients in this study were targeted for a low level of postoperative myopia at the patient’s request to overcome impending presbyopia. Therefore, the slightly lower efficacy index (0.94) compared to the other studies [11, 14, 24] may be attributed to a larger residual MRSE of − 1.14 ± 0.97 D at 12 months postoperatively. Regarding individual MRSE change, all eyes were within ± 1.00 D, and 89.2% were within ± 0.50 D from the 3 to 12 months follow-up period. The mean MRSE was rapidly stabilized at 1 day postoperatively and stably maintained up to 12 months after surgery (− 1.22 ± 1.26 D vs. − 1.14 ± 0.97 D, *P* = 0.830). The eyes with PRL implantation demonstrate excellent stability of the refractive correction during the entire follow-up period.

The early acute IOP elevations in the immediate postoperative period have rarely been studied in eyes with PRL implantation, however, possible similar mechanisms of elevated IOP could be seen with other pIOL implantation, such as ICL [18, 26, 27]. In this study, 37.4% (34/91) of the eyes suffered from early acute IOP elevations and required either close observation, topical antiglaucoma medications or anterior chamber drainage. Unlike the current version of the ICL, which has a central hole design and eliminates the need for a peripheral iridotomy [28–30], the PRL had no central hole and required preoperative laser peripheral iridotomies to prevent pupillary block. Consistent with ICL implantation [18, 26, 27], retained OVD was identified as the main cause of early acute IOP elevation in this study. The known possible mechanisms of IOP elevation after pIOL implantation included retained OVD, intraoperative anterior chamber overfilling, pupillary block, non-pupillary block angle closure, steroid response, pigment dispersion and malignant glaucoma etc. [26, 27, 31]. Among these, the most likely causes in our case series were retained OVD and intraoperative anterior chamber overfilling, because the IOP spike occurred within 1 day and resolved with non-invasive or minimally invasive treatment (close observation, anterior chamber drainage and/or topical antiglaucoma medications). This was confirmed by the observation of normal lens vaults and no evidence of pupillary block in these cases. Although overfilling of the anterior chamber cannot be completely ruled out, it was considered a less likely cause, assuming that the standard procedure of checking the IOP at the end of surgery was performed in all cases to ensure that an appropriate IOP was achieved. In this study, the OVD-free method did not eliminate but significantly decreased the incidence of early acute IOP elevations compared to the conventional method (28.8% vs. 53.1%, *P* = 0.022). However, the OVD-free method has been reported to completely eliminate the OVD-related IOP elevation for ICL implantation [18–20]. This discrepancy may be attributed to the small amount of OVD retained in the anterior chamber with PRL injection from the cartridge in the OVD-free method group (because PRL needs to be lubricated by OVD during injection in this study).

Endothelial cell loss with PRL implantation has been considered to be a result of the surgical manipulation rather than the presence of the PRL in the posterior chamber [9, 12, 32]. In this study, the initial mean endothelial cell loss was 11.3% at the 3-month postoperative visit with no further significant loss thereafter (9.6% at 12 months postoperatively, *P* = 0.395). This was consistent with previous studies [12, 32, 33] where a mild but significant loss of endothelial cell (4.6%–8.4%) was identified in the initial postoperative period due to the intraoperative manipulation and remained stable thereafter. The slightly higher initial endothelial cell loss in this study (11.3%) may be associated with certain intraoperative complications (PRL break, PRL flip) which required additional intraoperative manipulations. This was confirmed by observation where eyes that experienced intraoperative PRL break or flip had a higher incidence of notable endothelial cell loss > 20% (50.0% vs. 9.4% of the eyes,* P* = 0.005). Table 3 shows the association between intraoperative complications and notable endothelial cell loss, despite the surgical method used. Although this endothelial cell loss was not universal, there have been cases where intraoperative complications have occurred with only mild endothelial cell loss (Table 3, 001 OS, 009 OD, 042 OS, 053 OS). It was evident that intraoperative complications should be prevented to the greatest extent possible. PRL flip or break occurred in both groups with no statistically significant difference in incidence. Therefore, the occurrence of these complications was probably due to the inherent characteristics of the PRL, or the injection system rather than the surgical methods used. The PRL flip may be related to the rapid unfolding of the PRL, while PRL break may be associated with the fragility of the silicone material, exacerbated by an incompatible injector. However, even in eyes without intraoperative complications, 9.4% (9/96) experienced endothelial cell loss > 20% at the 3-month postoperative visit. This may be related to the unnoticed inadvertent contact of the PRL with the corneal endothelium during PRL unfolding. The PRL is made of silicone and unfolds rapidly. The injection system used in this study was also unable to control the rapid unfolding of the PRL in the anterior chamber, which increased the risk of contact between the PRL and the corneal endothelium. It is expected that the injection system will continue to be developed and improved to reduce this risk. Table 4 shows that eyes without intraoperative complications may also be at risk of notable endothelial cell loss with both surgical methods. This loss was defined as "notable" due to the belief that it may raise potential concerns about endothelium decompensation in the long term and requires close monitoring in these young patients. The surgical method may also affect endothelial cell loss for PRL implantation. The OVD-free method group had reached a borderline level of statistical significance (*P* = 0.056) with respect to endothelial cell loss compared with the conventional method group (9.4% vs. 13.6%), this may benefit from the elimination of the need to inject and remove the OVD, which can lead to a shorter operation time with less intraoperative manipulation [18–20].

The maintenance of an adequate lens vault was crucial for preventing crystalline lens contact or angle closure after pIOL implantation [34]. This may require an accurate prediction of the postoperative lens vault. However, due to the uncertainty in the lens vault associated with the floating design of the PRL, there is currently no formula available for predicting, and thus further investigation is warranted. Here, the lens vault continuously decreased during the 12-month postoperative period. This is consistent with Koivula's studies [12, 32], which found that the lens vault decreased significantly during the first year and stabilized thereafter. We found that the most remarkable changes in lens vault occurred within the first week after PRL implantation, with nearly half of the eyes (50.0%) having a decrease in lens vault and one tenth (10.4%) of the eyes having an increase. Additionally, 12.5% of the eyes had lens vault change greater than 200 μm. This rapid change of lens vault in the short-term postoperative period may correspond to the initial adaptation of the PRL to the configuration of the anterior segment of the eye. However, the process of PRL stabilization in the posterior chamber continued up to 1 year postoperatively with lens rotation to be observed in the majority of the eyes [12, 32]. With the continuous decrease of lens vault, four eyes of two patients in this study developed PRL and crystalline lens contacts (lens vault less than 10 μm) with no sign of cataract formation up to 1 year postoperatively. The contact between the pIOL and the anterior surface of the crystalline lens [9, 35], as well as the reduction in the circulation of aqueous humor to the anterior surface of the crystalline lens [36, 37] have been considered as the two main factors causing secondary cataract. The aqueous humor circulation was improved in the later version of ICL with a central hole [28, 29, 38] and this modification may potentially reduce the risk of developing cataract [39, 40]. Although the current model of PRL did not have a central hole design, a low incidence (0%–2.86%) of cataract formation after PRL implantation has been reported in the literature [11, 12, 14, 24, 32, 33, 41]. This may benefit from the rotating and floating design of the PRL which protects the crystalline lens from continuous contact with the implanted PRL [10, 12, 24]. Portaliou et al. [41] reported the long-term results of a retrospective series of 143 eyes implanted with PRL, 34 of these eyes were followed for 6 years, during which time no signs of cataract formation were observed. Pérez-Cambrodí et al. [24] reported that only 1 eye out of 35 (2.86%) developed cortical lens opacity during a mean follow-up of 57.34 ± 9.24 months. Torun et al. [42] reported that in 53 eyes with a mean follow-up of 86 ± 21.2 months, four eyes (7.5%) developed clinically significant cortical lens opacities requiring cataract surgery. In our study, the observed lenticule changes, increased lens density (patient 10) and punctate lens opacities in the periphery (patients 09 and 24), appeared to be non-specific for PRL-related issues, as anterior subcapsular cataract was considered the most common type of posterior chamber pIOL-induced cataract [35, 43], and patient-dependent factors such as age (ages 43–46 years) and refractive status (− 14.0 D to − 22.75 D) may also play a role [34, 35, 43]. Although no clinically significant cataract formation was found over the 12-month follow-up in this study, it is essential to continue monitoring the encouraging short-term results with a more prolonged study to assess long-term lenticule changes following PRL implantation.

Ideally, the PRL is expected to float freely within the posterior chamber space and the flexible haptics should rest on the zonule without causing any damage [9]. However, this is not always the case in practice and the previous study [44] has reported that only 37.5% (6/16) of PRLs were in the ideal position with both haptics on the zonule. The significant variability in haptic location has raised concerns about the size selection strategy for PRL implantation. In our study, the size selection was still based on the white-to-white distance, as recommended by the manufacturer, and was limited to only two available sizes for myopic eyes (BK 108 and BK 113). Although the short-term observation (12 months) revealed only mild PRL decentrations in seven eyes and no pupillary block or lens subluxation were noted, further investigation of the intraocular interaction of the PRL with posterior chamber structures using appropriate technology, such as ultrasound biomicroscopy [44], was warranted to determine the suitability of the PRL size. In the future, lens size should be selected according to a more detailed characterization of the posterior chamber space and, if possible, more size options are expected to be available. It is noteworthy that unsuitable PRL size may potentially cause delayed zonular dehiscence with PRL luxation [45–47]. This severe complication was thought to be associated with progressive weakening of the zonules, which may be caused by continuous excessive pressure of haptics against zonules [9, 11]. The high degree of myopia is a known risk factor for having preexisting zonular weakness [9, 15, 16]. In our study, nearly two-thirds of the eyes had extremely high myopia (less than or equal to − 12.00 D) and postoperative complications were monitored for 1 year. Although no PRL luxation was observed and the short-term safety of PRL implantation for extremely high myopia was preliminarily validated, continuous monitoring for this potential complication was still necessary.

In previous studies [18–21], OVD-free methods, although may use different surgical techniques or instruments, were initially proposed to intentionally eliminate OVD-related complications and simplify the surgical procedure. This has been achieved in our previous study for ICL implantation without causing additional complications[18]. Based on the promising results with ICL, it was reasonable to use this technique for PRL implantation, which shared several similar features with ICL, as both were implanted in the posterior chamber through a corneal incision. In this study, the OVD-free method with patent irrigator was identical to the previously reported technique [18], and its safety and efficacy with PRL implantation were fully investigated by comparing intraoperative complications, postoperative complications, postoperative IOP, and endothelial cell loss between two groups. As previously stated, the OVD-free method resulted in a significant reduction in the incidence of early acute IOP elevations (*P* = 0.022) and potentially caused less initial endothelial cell loss (*P* = 0.056) compared with the conventional method. More importantly, this benefit was achieved without increasing either intraoperative or postoperative complications (Table 5). It is worth noting that three eyes (4.8%) that experienced PRL break or flip, which required PRL explantation and re-implantation, were converted intraoperatively from the OVD-free method to the conventional method. These method conversions were done for the sake of maximum safety as the OVD could maximize the stability of the anterior chamber during the PRL explantation [48]. Furthermore, it should be carefully evaluated in different cohorts of patients to validate the safety and efficacy of the OVD-free method. The design of this study was inherently limited by its real-world nature. Prospective noninterventional settings allow patients to be followed under real-world conditions, and thus collecting valuable data without the confounding effects of interventions such as a randomized controlled trial that would provide a higher level of evidence. With the promising results in this study, we are encouraged to, if possible, conduct a randomized controlled trial in the future. As the technique continues to be refined and its benefits further validated, it is appealing to speculate on the potential future applications of the OVD-free method in other types of intraocular refractive surgery.

A major limitation of this study was the relatively high rate of loss to follow-up at 6 months and 12 months postoperatively due to the real-world, noninterventional setting and lockdown during the COVID-19 pandemic in China. This could potentially compromise the study's validity as an incomplete database was used. To address this issue, this study used linear mixed models for statistical analysis, which was found to be highly effective in handling missing values compared to the conventional method of using Analysis of Variance. The linear mixed models allowed us to maximize the usefulness of the available data, despite the limitation of incomplete data. Another limitation to highlight was the inclusion of bilateral eyes, which may introduce a potential confounding factor affecting the validity and generalizability of our results. The break rate observed in this study, with seven cases of PRL break in 108 eyes (6.5%), was indeed a notable finding that warrants attention. This was the first report on the incidence rate of PRL break, it may be due to the fragility of the silicone material, exacerbated by an incompatible injector. Efforts should be made to develop injection systems that are more compatible with PRL properties to ensure safer and more efficient implantation procedures.

## Conclusions

PRL implantation was a safe, efficient, predictable and stable method of correcting high myopia. No clinically significant cataract formation or PRL luxation were observed for up to 12-month follow-up, however, continuous monitoring for these potential complications was still necessary. The injection system is particularly important for PRL implantation, and its improvement is expected to further reduce intraoperative complications. The OVD-free method is efficient for PRL implantation in highly myopic eyes without increasing either intraoperative or postoperative complications. The potential of lower incidence of early acute IOP elevations makes the OVD-free method a promising alternative to the conventional method and deserves careful evaluation in different cohorts of patients to confirm its values for PRL implantation.

## Data Availability

The datasets used and/or analyzed during the current study are available from the corresponding author on reasonable request.

## Abbreviations

pIOL: Posterior chamber phakic intraocular lens
ICL: Implantable collamer lenses
D: Diopters
PRL: Phakic refractive lens
IOP: Intraocular pressure
OVD-free: Ophthalmic viscosurgical device-free
MRSE: Manifest refraction spherical equivalent
K: Keratometry
ACD: Anterior chamber depth
CDVA: Corrected distance visual acuity
UDVA: Uncorrected distance visual acuity
ECD: Corneal endothelial cell density
AL: Axial length
OVD: Ophthalmic viscosurgical device
MPE: Mean prediction error
MAE: Mean absolute error
RMSE: Root mean square error
